# Functional Characterization of the 14-3-3 Gene Family in Alfalfa and the Role of *MsGRF2* in Drought Response Mechanisms

**DOI:** 10.3390/ijms252212304

**Published:** 2024-11-16

**Authors:** Lu Chai, Yuxuan Liu, Jiuding Sun, Xinhang Duan, Mei Yang, Kailin Qian, Pan Zhang

**Affiliations:** College of Animal Science and Technology, Northeast Agricultural University, Harbin 150030, China; chailu0919@163.com (L.C.); 13634825658@163.com (Y.L.); 15004621196@163.com (J.S.); duanxinhang96@163.com (X.D.); yangmei199611@163.com (M.Y.); 18800469076@163.com (K.Q.)

**Keywords:** *Medicago sativa* L., antioxidant enzymes, G-box Regulatory Factor, stress response mechanisms, abiotic stress

## Abstract

Drought stress affects crop growth and development, significantly reducing crop yield and quality. Alfalfa (*Medicago sativa* L.), the most widely cultivated forage crop, is particularly susceptible to drought. The general regulatory factor (GRF) protein 14-3-3, a highly conserved family in plants, specifically recognizes and binds to phosphoserine residues in target proteins, regulating both plant development and responses to environmental stressors. In this study, 66 alfalfa 14-3-3 proteins were identified, and the full-length *MsGRF2* gene was cloned and functionally analyzed. The expression of *MsGRF2* was highest in alfalfa inflorescences and lowest in roots. Transgenic tobacco overexpressing *MsGRF2* exhibited increased tolerance to low temperature and drought stress, evidenced by physiological indicators including low levels of active oxygen species and increased activity of antioxidant enzymes and osmoregulatory substances. Under drought stress conditions, compared to wild-type plants, *MsGRF2*-overexpressing tobacco plants exhibited significantly increased expression of drought stress-related genes *ERD10B* and *TIP*, while the expression of *BRI1*, *Cu*/*Zn-SOD*, *ERF2*, and *KC1* was significantly reduced. Together, these results provide new insights into the roles of the 14-3-3 protein MsGRF2 in plant drought response mechanisms.

## 1. Introduction

Drought is a major climatic challenge, impacting environmental stability and limiting global agricultural productivity [1,2], with around 35% of the world’s land facing intensified drought conditions [3]. Drought stress adversely affects plant growth, development, and productivity by altering growth rates; inhibiting root, shoot, and leaf expansion; and impairing seed germination and seedling growth, which ultimately leads to abnormal development and reduced biomass [4,5]. Prolonged drought exacerbates oxidative stress in plants, leading to significant declines in plant vigor or even plant death [6]. Plants use enzymatic and non-enzymatic antioxidant systems to defend against drought-induced oxidative stress [7], where the activation of the reactive oxygen species (ROS) scavenging system mitigates oxidative damage and enhances drought tolerance by balancing ROS production and scavenging [8]. Drought stress disrupts the optimal water status in plants, posing a challenge to their survival. Maintaining this optimal water balance is crucial for plants under drought conditions, with osmotic adjustment (OA)—the accumulation of compatible solutes—serving as a primary adaptive response to mitigate water deficit [9]. OA facilitates a net increase in osmotically active solutes within cells, which lowers osmotic potential, enhances cellular hydration, and supports the expansion of leaves and other metabolically active cells [10].

The 14-3-3 protein is an acidic, soluble, heterodimeric protein that recognizes and binds phosphorylated target proteins, thereby modulating their stability and activity [11]. Beyond this role, 14-3-3 proteins regulate various biological processes and participate in signal transduction by modulating the activities of their target proteins [11]. The 14-3-3 protein has been identified across various plant species, including *Arabidopsis thaliana*, where 13 distinct 14-3-3 proteins have been categorized into two groups: five belonging to the ε group and eight to the non-ε group [12]. In *A. thaliana*, 14-3-3 proteins, also known as GF14 (G-box Factor 14-3-3 homologs) or GRF (G-box Regulatory Factor/General Regulatory Factor) proteins, function as components of the G-BOX complex and are designated as GRF1-13, and specifically as GRF1 (14-3-3χ), GRF2 (14-3-3ω), GRF3 (14-3-3ψ), GRF4 (14-3-3Φ), GRF5 (14-3-3υ), GRF6 (14-3-3λ), GRF7 (14-3-3ν), GRF8 (14-3-3κ), GRF9 (14-3-3μ), GRF10 (14-3-3ε), GRF11 (14-3-3ο), GRF12 (14-3-3ι), and GRF13 (14-3-3π) [12]. In other plant species, 14-3-3 proteins have been identified in the following quantities: eight in *Oryza sativa* L., 10 in *Medicago truncatula*, 28 in *Zea mays* L., six in *Sorghum bicolor* (L.) Moench, seven in *Phragmites australis* (Cav.) Trin. ex Steud., and 18 in *Solanum tuberosum* L. [13,14].

The 14-3-3 protein is involved in various metabolic pathways in plants and regulate growth and developmental processes [15]. For instance, in *A. thaliana* and *O. sativa*, the 14-3-3 protein family negatively regulates brassinosteroid signaling by anchoring *BRZ1* and *BRZ2*/*BES1* transcription factors in the cytoplasm [16]. In *Spinacia oleracea* L., 14-3-3 proteins interact with serine (Ser) 229 residue of sucrose–phosphate synthase (SPS) [17], an enzyme known for its crucial role in osmotic stress [18]. In *O. sativa*, the 14-3-3 protein GF14f regulates seed development by modulating the activity of sucrose synthase (SuSase), adenosine diphosphate-glucose pyrophosphorylase (AGPase), and starch synthase (StSase) in seeds [19,20]. In *A. thaliana*, 14-3-3 proteins interact with transcription factor 1 (*WRI1*) to mediate seed oil content [21]. The 14-3-3 proteins also modulate roles in stress response [22]. For example, *MdGRF2* encodes a 14-3-3 protein that enhances salt and drought tolerance in *Malus domestica* (Suckow) Borkh. [23], while, *BdGF14d* encodes a 14-3-3 protein that enhances salt tolerance in *Nicotiana tabacum* L. (tobacco) via the ABA signaling pathway, ROS scavenging, and ion transporting channels [24]. Another key function of 14-3-3 proteins in plant stress response is their interaction with other proteins involved in stress signaling pathways. For example, 14-3-3 proteins can directly or indirectly synergize with calcium-dependent protein kinases (CDPKs) to modulate plant physiological responses, thereby enhancing adaptive responses to environmental stresses [25].

Alfalfa (*Medicago sativa* L.), often known as the “king of forage”, is a perennial legume of significant economic and social value due to its high nutritional content and adaptability [26]. Although alfalfa exhibits relatively better drought tolerance than many other forage crops, it faces increasing challenges from intensifying and prolonged drought conditions driven by climate change [27]. While the role of 14-3-3 proteins in plant stress responses is well-documented, there remain substantial gaps in our understanding of how they confer drought tolerance in alfalfa. This study aims to identify genes encoding 14-3-3 proteins (hereinafter referred to as *MsGRF*) in alfalfa and to characterize their role in enhancing drought tolerance. By clarifying the regulatory function of 14-3-3 proteins in drought response, this study seeks to provide insights that could inform the development of drought-resistant alfalfa cultivars.

## 2. Results

### 2.1. Identification of GRF Gene Family Members and Chromosomal Localization

**Figure 1:**
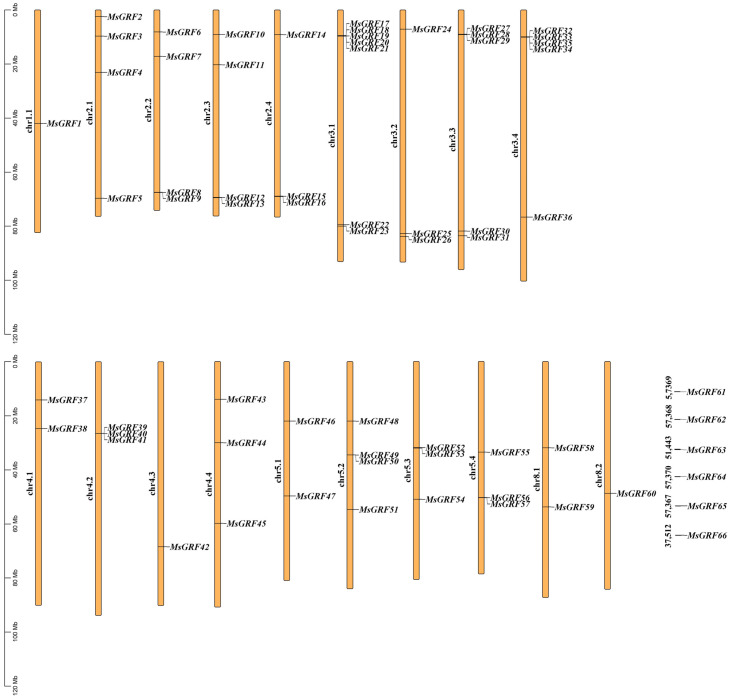
Chromosomal localization of GRF gene family members in *M. sativa*. *MsGRFs* genes were unevenly distributed across the 19 chromosomes of *M. sativa*.

A total of 87 *MsGRF* candidate genes were initially identified through HMM searches using the 14-3-3 structural domain (PF00244). Additionally, BLAST analysis based on the *A. thaliana* 14-3-3 gene family identified 66 *MsGRF* candidate genes. Intersection of these two datasets yielded 66 *MsGRF* genes. Chromosomal localization analysis of the 66 MsGRFs identified them as MsGRF1-66 based on their chromosomal positions. The genes were mapped along 19 chromosomes, with 13 chromosomes lacking any *MsGRF* genes and six MsGRFs remaining unlocalized on chromosomes (Figure 1).

### 2.2. Phylogenetic Analysis and Classification of GRF Gene Family Members

**Figure 2:**
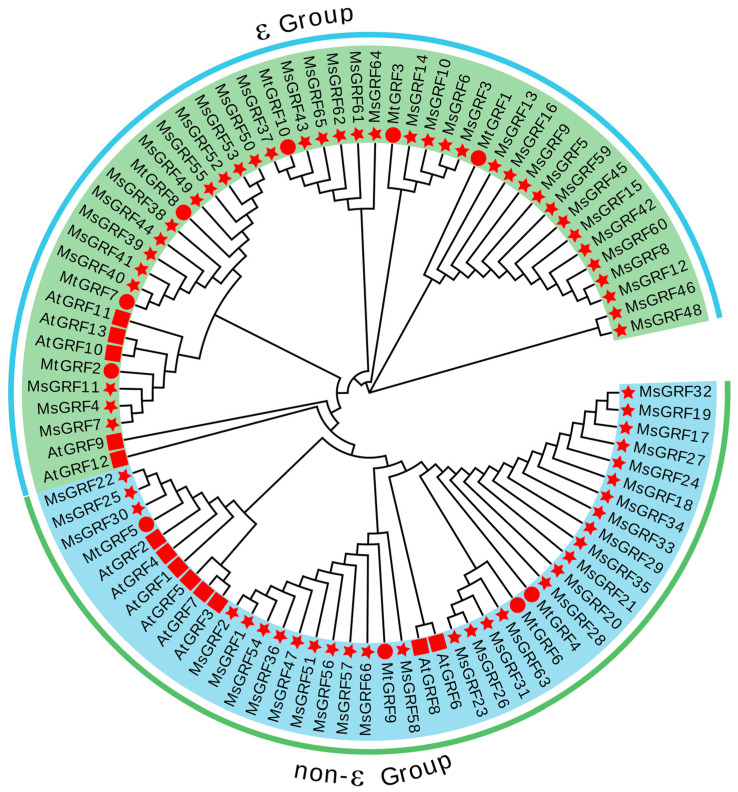
Phylogenetic analysis of GRF gene family members in *M. sativa*, *A. thaliana,* and *M. truncatula*. The neighbor-joining tree was generated using MEGA 7 with 400 bootstrap replicates. Different subgroups are indicated by different colors. Red circles indicate *M. sativa* GRF proteins.

Phylogenetic analysis was conducted for the 13 *A. thaliana AtGRFs*, 10 *M. truncatula MtGRFs*, and 66 *M. sativa MsGRFs* identified here (Figure 2). The 89 GRFs were classified into two sub-groups: the ε and non-ε groups. Groups 1 and 2 each contained 21 GRFs, with ε group containing five AtGRFs, six MtGRFs, and 36 MsGRFs. The non-ε group contained eight AtGRFs, four MtGRFs, and 30 MsGRFs, consistent with observations in *M. truncatula* and *A. thaliana*.

### 2.3. Physical and Chemical Properties of MsGRF Proteins

The ExPASY platform was used to analyze the physical and chemical properties of MsGRF proteins (Table S1), revealing considerable differences among protein types. MsGRF48, encoding 924 amino acids, exhibited the highest molecular weight at 33.35 kDa, while MsGRF36, encoding 258 amino acids, had the lowest molecular weight at 9.84 kDa. The protein hydrophobicity index ranged from −0.643 (observed in MsGRF3, MsGRF6, MsGRF10, and MsGRF14) to −0.173 (MsGRF36), indicating that all MsGRFs are hydrophilic. The theoretical isoelectric points of the proteins varied between 4.68 (recorded for MsGRF23, MsGRF36, MsGRF31, MsGRF63) and 7.68 (MsGRF8), with 65 (98.5%) MsGRFs classified as acidic (pI < 6.5), and only one (1.5%) classified as basic (pI > 7.5).

### 2.4. Domain, Motif, and Gene Structure Analysis of MsGRFs

**Figure 3:**
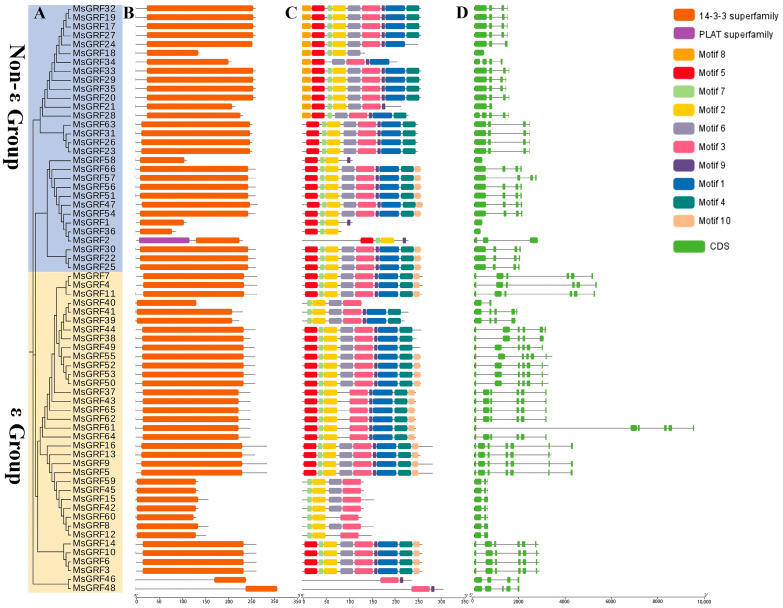
(**A**) Phylogenetic analysis of MsGRFs. (**B**) MsGRF domains. (**C**) Motif details for the identified conserved domains of MsGRF proteins. Each motif is represented by a different colored box. (**D**) The gene structures of candidate *M. sativa* GRF genes. Green boxes indicate CDS, and black lines indicate introns. The number shows the phases of the relevant intron.

A phylogenetic tree was constructed for the 66 *MsGRFs*, categorizing them into two distinct groups: 30 *MsGRF* genes in the non-ε group contained four exons and three introns, while those in the ε group generally contained 6–7 exons and 4–6 introns (Figure 3A,D). Protein domain, motif, and gene structural analyses were also conducted. A conserved structural protein domain analysis indicated that all MsGRFs possessed the 14-3-3 protein structural domain (Figure 3B). Motif analysis revealed that members of the same group shared structural similarities; for instance, *MsGRF34* lacked motif2 and motif7, whereas some MsGRFs in the first group contained motif8, but lacked motif10. The motifs 5, 7, 2, 6, 3, 9, 1, and 4 were relatively conserved among the *MsGRFs*, likely corresponding to components of the 14-3-3 superfamily domain (Figure 3C and Figure S1, Table S2).

### 2.5. Analysis of Cis-Acting Elements of MsGRF Promoters

**Figure 4:**
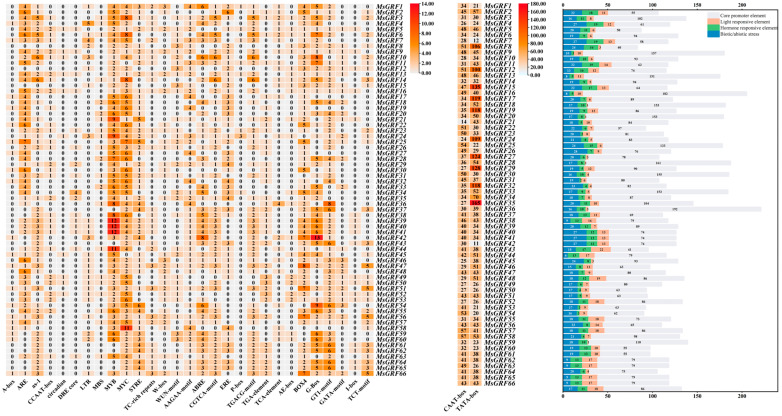
Promoter analysis of MsGRFs. The number of cis-acting elements are shown in the grid with different numbers and colors. The sums of the cis-acting elements across the four classes of each gene are shown in the histogram.

The sequences of the 66 *MsGRFs* genes, including 2000 bp upstream of the coding region (CDS), were extracted using TBtools, and their promoter cis-acting elements were analyzed. These elements were classified into three categories: stress-associated, hormone-associated, and light-responsive regulatory elements. Among the *MsGRF* genes, *MsGRF25* contained the highest number of stress-related components (28), while *MsGRF43* had the fewest (6). For hormone-associated cis-acting elements, *MsGRF10* exhibited the highest count (19), while *MsGRF3* had the lowest (2). Additionally, *MsGRF35* contained the greatest number of core elements, including TATA-box and CAAT-box motifs (192) and had the highest overall elements (221) (Figure 4). Notably, stress-responsive elements were dominated by MYB and MYC, hormone-responsive elements by ABRE, and light-responsive elements by G-BOX.

### 2.6. Collinearity Analysis of MsGRF Genes

**Figure 5:**
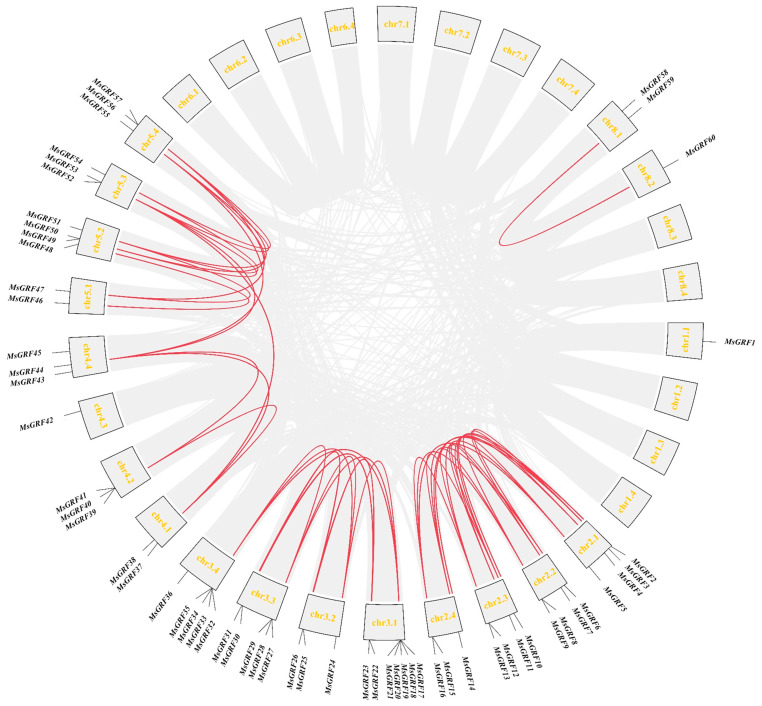
Synteny analysis of *MsGRF* genes in *M. sativa*. Gray lines indicate whole-genome synteny blocks, while red lines indicate replicated MsGRF gene pairs.

**Figure 6:**
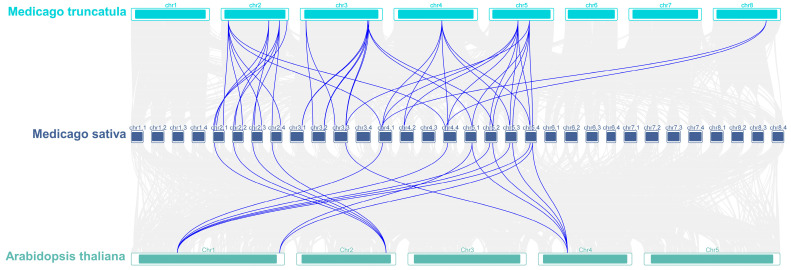
Collinearity analysis of GRF genes in *M. sativa*, *A. thaliana*, and *M. truncatula*. Gray lines in the background indicate collinear blocks within *M. sativa* and other plant genomes, while blue lines indicate syntenic GRF gene pairs.

A total of 54 pairs of collinear events were identified among 46 MsGRFs, including 15 one-to-one, 12 one-to-two, and five one-to-three collinear relationships, potentially linked to large segmental duplication events. The existence of 13 tandem repeats indicates that gene duplication event occurred during the evolution of MsGRFs, driving the rapid expansion and high level of homology within the MsGRF gene family (Figure 5). To further explore the evolutionary relationships of the GRF gene family across plant species, a collinearity analysis of GRF genes in *M. sativa*, *M. truncatula* and *A. thaliana* was conducted. Sixteen MsGRFs exhibited collinear relationships with *A. thaliana* and 42 displayed collinear relationships with *M. truncatula* (Figure 6).

The non-synonymous (Ka) and synonymous (Ks) substitution rates of each collinear gene pair were then calculated. The Ka/Ks rations for 49 MsGRF gene pairs were found to be greater than 1, indicating purifying selection, whereas seven MsGRF gene pairs were had Ka/Ks ratios greater than 1, suggesting they had undergone positive selection (Table S3).

### 2.7. Characterization and Isolation of MsGRF2

**Figure 7:**
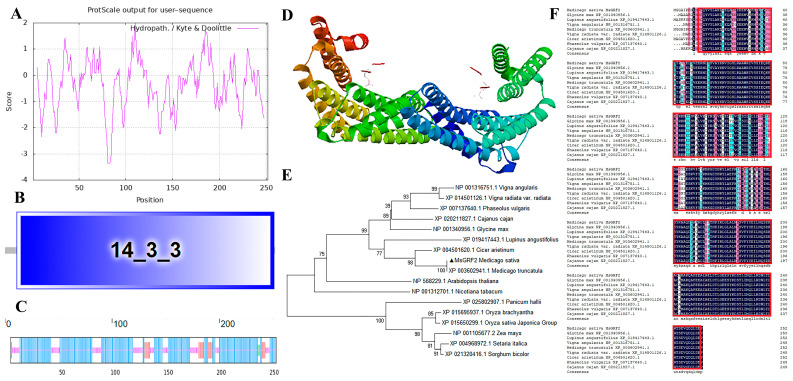
Tertiary-level structure, phylogenetic analysis, and amino acid sequence alignment of MsGRF2 proteins. (**A**) MsGRF2 Protein hydrophilicity and hydrophobicity prediction. (**B**) Domains of MsGRF2 protein (**C**) Secondary structure for MsGRF2 protein. (**D**) 3D protein model of MsGRF2. (**E**) Phylogenetic tree of MsGRF2. (**F**) Amino acid sequence alignment.

Given that the results of this study identified MsGRF2 as the only protein possessing a PLAT domain, a feature associated with abiotic stress responses, we further investigated and characterized the potential role of MsGRF2 in response to abiotic stress in alfalfa. *MsGRF2* has a CDS length of 759 bp, encoding 252 amino acids. The molecular formula of MsGRF2 is C_1255_H_1983_N_327_O_406_S_9_, with a theoretical isoelectric point of 4.68. The calculated weight of MsGRF2 protein is 28.4 kD, containing 20 basic amino acids, with an instability coefficient of 44.32, indicating it is unstable. The MsGRF2 has a fat coefficient of 90.6, and an average hydrophilicity coefficient of −0.37, suggesting it is hydrophilic (Figure 7A). SMART domain analysis revealed a 14-3-3 structural domain from 11–251 bp, classifying MsGRF2 as a member of the 14-3-3 family (Figure 7B). Secondary structure analysis showed that MsGRF2 consists of69.05% α-helices, 6.75% β-extended strands, 1.19% β-turned corners, and 23.02% random coils (Figure 7C).

A phylogenetic analysis confirmed that MsGRF2 exhibited significant conservation across leguminous and gramineous species, particularly showing high homology with *Tribulus terrestris*, indicating potential functional similarity (Figure 7E). Homology analysis compared *MsGRF2* with genes from *M. truncatula*, *Glycine max*, *Lupinus angustifolius*, *Vigna angulsris*, *V. radiata var*, *Radiata*, *Cicer arietinum*, *Phaseolus vulgaris*, and *Cajanus cajan,* revealing sequence similarities of 100.00%, 88.49%, 88.10%, 86.11%, 86.90%, 95.63%, 86.11%, and 84.92%, respectively (Figure 7F). These results indicate that MsGRF2 may be highly conserved across species, suggesting evolutionary conservation at the molecular level.

### 2.8. Subcellular Localization and Expression Patterns of MsGRF2

**Figure 8:**
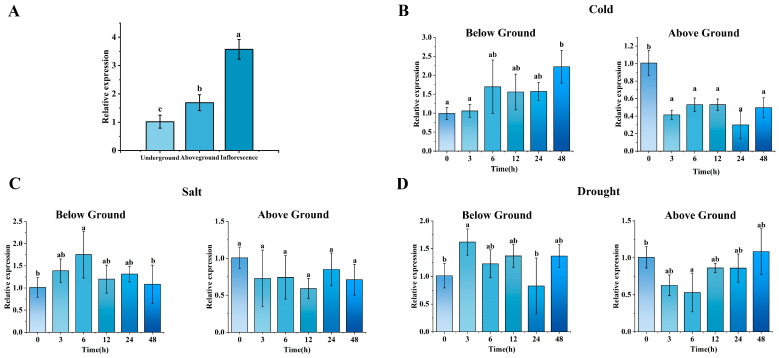
(**A**) qRT-PCR (quantitative real-time PCR) analysis of *MsGRF2* expression levels in different alfalfa tissues (above-ground, below-ground, and inflorescence tissues). Expression patterns of *MsGRF2* observed in four-week-old self-rooted alfalfa seedlings after treatment with ddH_2_O, (**B**) low temperature (4 °C), (**C**) PEG6000, and (**D**) 150 mM NaCl for 0, 3, 6, 12, 24, and 48 h after treatment, respectively. Error bars represent means ± SD (n = 3) from three independent biological replicates. Different letters indicate statistically significant differences (Duncan’s tests, *p* < 0.05).

To assess the subcellular localization of MsGRF2, the expression vectors 35S::MsGRF2-sGFP and 35S::sGFP were introduced into tobacco (*Nicotiana benthamiana*) leaves (Figure S2). The results demonstrated that MsGRF2 was localized in the nucleus, cytoplasm, and cell membrane (Figure S3). An analysis of expression patterns under non-stress conditions revealed that *MsGRF2* was particularly expressed in above-ground tissues and inflorescences of alfalfa (Figure 8A). The expression level of *MsGRF2* was highest in inflorescences, significantly exceeding those in above-ground and below-ground tissues. Conversely the lowest expression level was recorded for below-ground tissues, with significantly reduced levels compared to both inflorescences and above-ground tissues (*p* < 0.05).

The pattern of *MsGRF2* expression was investigated under cold stress (4 °C), drought conditions (20% PEG6000), and salt stress (150 mM NaCl). Under cold stress, *MsGRF2* expression in above-ground tissues was significantly down-regulated, reaching its lowest level at 24 h after treatment (HAT), whereas expression in below-ground tissues was highest at 48 HAT, and significantly higher than the control group (*p* < 0.05) (Figure 8B). Salt stress did not significantly affect *MsGRF2* expression in above-ground tissues; however, in below-ground tissues, expression peaked at 6 HAT, significantly higher than the control group (*p* < 0.05) (Figure 8C). Under drought treatment, *MsGRF2* expression in above-ground tissues initially decrease, reaching a minimum at 6 HAT, followed by an increase over time. This lowest expression was significantly lower than the control group (*p* < 0.05). In contrast, *MsGRF2* expression in below-ground tissues reached peaked at 3 HAT, significantly higher than the control and other time points (*p* < 0.05), with a subsequent decline at 6 HAT, which did not differ significantly from the control (Figure 8D).

### 2.9. Transformation of MsGRF2 and Screening of Tobacco Plants

**Figure 9:**
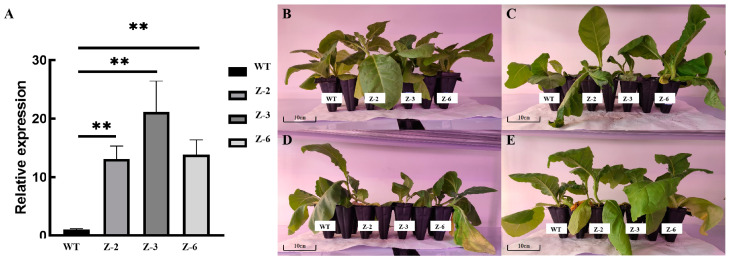
(**A**) Relative expression levels of *MsGRF2* in transgenic tobacco and transgenic tobacco clone plants treated with (**B**) control conditions, (**C**) 4 °C, (**D**) 20% PEG6000, and (**E**) 150 mM NaCl. The scale bar equals 10 cm. Different symbols show statistically significant differences (*t*-test; **: *p* < 0.01).

To explore the function of *MsGRF2*, it was introduced into tobacco plants (Figure 9A). Under control conditions, transgenic tobacco lines Z-2, Z-3, and Z-6 exhibited enhanced leaf growth compared to the wild-type (WT) (Figure 9B), indicating that *MsGRF2* promoted plant growth. Under cold stress, both transgenic tobacco and WT plants display damage, indicating that *MsGRF2* does not confer cold tolerance (Figure 9C). Under drought conditions, WT leaves exhibited wilting, while transgenic plants showed no notable stress symptoms (Figure 9D), suggesting that *MsGRF2* enhanced drought tolerance. Under salt stress, no significant obvious physiological differences were not observed between WT and transgenic plants (Figure 9E). Overall, these results suggest that *MsGRF2* may primarily play roles in mitigating drought stress.

### 2.10. Overexpression of MsGRF2 Increased Sensitivity to Drought Stress in Transgenic Tobacco Plants

**Figure 10:**
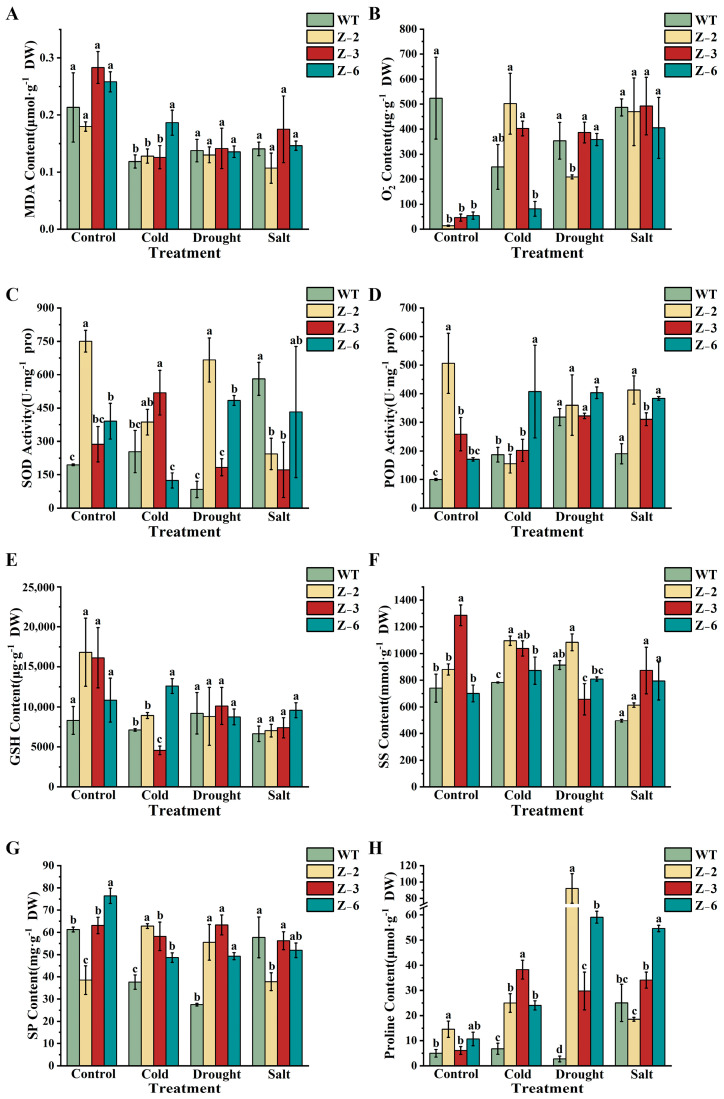
Physiological indices of control and transgenic line plants after cold, drought, and salt stress conditions after 5 days. Measured indices include the levels of (**A**) malondialdehyde (MDA), (**B**) superoxide anion (O_2_^−^), (**C**) superoxide dismutase (SOD), (**D**) peroxidase (POD), (**E**) glutathione (GSH), (**F**) soluble sugar (SS), (**G**) soluble protein (SP), and (**H**) proline (Pro). Different letters show statistically significant differences.

To gain insights into the physiological basis of the various responses observed in transgenic plants under different stress conditions, several stress-associated physiological indicators, including malondialdehyde (MDA), superoxide anion (O_2_^−^), superoxide dismutase (SOD), peroxidase (POD), glutathione (GSH), soluble protein (SP) contents, soluble sugar (SS) contents, and proline (Pro) contents were evaluated (Figure 10). In transgenic plants, the levels of all indicators were generally equal to or greater than those in WT plants, except for O_2_^−^, which was reduced, suggesting that elevated *MsGRF2* expression reduces O_2_^−^ accumulation. No significant differences in MDA and GSH contents were observed between WT and transgenic plants under drought stress (Figure 10A, E). The SOD levels were higher in transgenic plants compared to WT, though no significant differences were recorded under salt and cold stress treatments (Figure 10C). The SS and SP levels of transgenic plants were higher than those in WT plants under cold and drought stress treatments (Figure 10F,G). The Pro levels were consistently and significantly higher in transgenic plants across all stress conditions (Figure 10H), indicating a critical role for proline in the stress response mechanism mediated by MsGRF2. Overall, these physiological indicators confirm that *MsGRF2* enhances abiotic stress tolerance by promoting the accumulation of specific protective compounds, though this gene may have limited efficacy in conferring tolerance to cold and salt stress.

**Figure 11:**
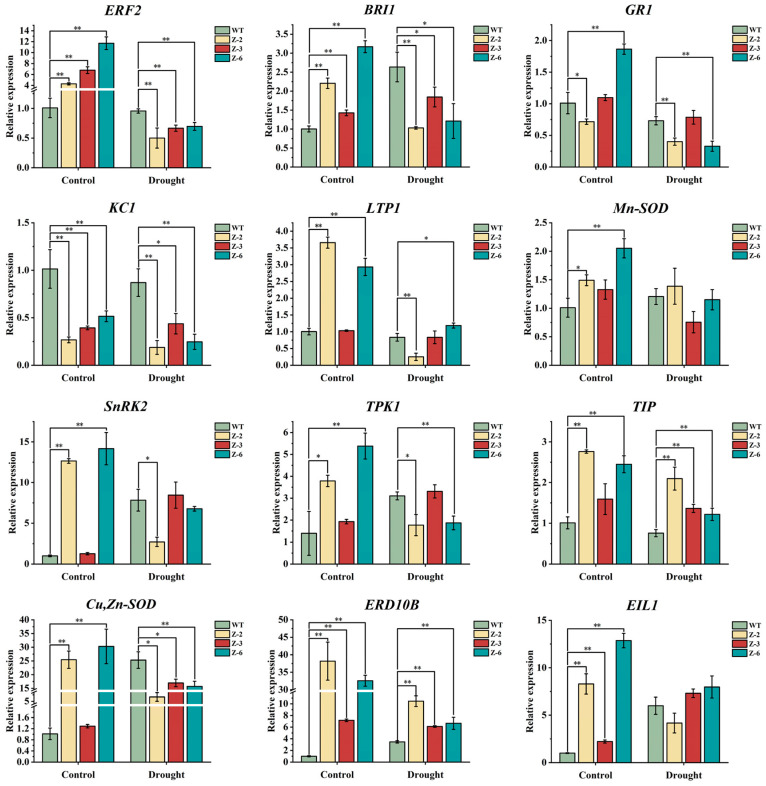
Expression of important marker genes in the WT and transgenic lines under drought stress after 5 days. qRT-PCR results are shown for the stress-related genes *BRI1*, *Cu*, *Zn-SOD*, *Mn-SOD*, *EIL1*, *ERF2*, *GR1*, *ERD10B*, *TIP*, *LTP1*, *SnRK2*, *KC1,* and *TPK1* in tobacco seedlings after drought treatment. Error bars show the means ± SD (n = 3) from three independent biological replicates. Different letters show statistically significant differences (*t*-test; *: *p* < 0.05; **: *p* < 0.01).

Based on the physiological indicator analyses, the drought treatment was selected for subsequent experiments. To explore the underlying the mechanisms through which *MsGRF2* enhances stress tolerance, the expression levels of stress-responsive and ROS scavenging genes in WT and transgenic plants were investigated. The genes investigated included *BRI1*, *Cu*, *Zn-SOD*, *Mn-SOD*, *EIL1*, *ERD10B*, *ERF2*, *GR1*, *LTP1*, *KC1*, *SERK3B*, *SnRK2*, *TIP,* and *TPK1*. qRT-PCR analyses indicated significant up-regulation of all genes under control conditions except for *KC1*. Under drought stress, *BRI1*, *Cu*/ *Zn-SOD*, *ERF2*, *KC1,* and *TPK1* were down-regulated, while *ERD10B* and *TIP* were up-regulated (Figure 11). These expression patterns suggest possible interaction mechanisms among these genes in response to drought stress. Overall, *MsGRF2* expression modulated the transcription of key stress-related genes, contributing to enhanced drought tolerance in transgenic plants.

## 3. Discussion

Drought severely impairs plant growth and development, making it essential to enhance drought tolerance in crops to improve yield, quality, and economic value [28,29]. The 14-3-3 gene family is recognized for its roles in abiotic stress responses across various crops [30,31]. However, its specific function in conferring stress tolerance in alfalfa, along with the molecular mechanisms underlying this tolerance, remains unclear. In this study, we identified a total of 66 MsGRF family members in the genome of alfalfa, which is considerably greater than those identified in *A. thaliana* (15) [32], *Oryza sativa* (8) [33], *Capsicum annum* (15) [34], *Solanum lycopersicum* (12) [35], *Brachypodium distachyon* (12) [36], *G. max* (18) [37], and *M. domestica* (18) [38]. This result suggests that the 14-3-3 gene family has undergone significant evolutionary development in alfalfa. Notably, it was found that MsGRF61–MsGRF66 were not chromosome-anchored, while the remaining 60 MsGRF members were unevenly distributed across 19 chromosomes, with each chromosome containing 1–7 genes (Figure 1). Tandem and segment duplication events are known to drive gene family expansion during plant evolution [39,40]. In this study, we identified 54 segmental repeats and 13 tandem repeats among the MsGRF genes, underscoring the role of segmental duplications in the evolution of this gene family (Figure 5).

All 66 MsGRF members contain conserved domains characteristics of the 14-3-3 protein family (PF00244), with an average molecular weight of 26.52 kD, approximately30 kD lower than other GRF proteins, and an average pI of 5.03 (Table S1). The acidic pI value recorded for the MsGRF in this research is similar to those recorded for GRF proteins in other species, although some, such as MsGRF8, exhibit basic properties [13,33,37,41]. SMART analysis confirmed that MsGRF2 contains a highly conserved domain, and phylogenetic analysis revealed MsGRF clustered into two distinct clades—one clustering with leguminous plants and the other with gramineous species—indicating a high degree of evolutionary conservation within the 14-3-3 protein family, which is in agreement with findings from previous studies [33,41,42]. Phylogenetic analysis also indicated that 54.5% of MsGRF family members belong to ε-type, while 45.5% are non-ε-type, consistent with the hypothesis that states GRF proteins evolved into two groups at an early stage of evolution [43]. An analysis of cis-elements revealed that the promoter regions of the *MsGRF* genes contain abundant cis-elements associated with hormone responses (e.g., abscisic acid and jasmonic acid), stress responses (e.g., low temperature and drought), and light responses, suggesting hormone- and stress-responsive regulatory roles for these genes [36,44]. Similar stress-responsive behavior of GRF proteins has been observed in other plants [34,38,45]. In this study, we identified that MsGRF2 was the only GRF protein among the 66 detected in alfalfa to possess a PLAT domain, a protein domain associated with abiotic and biotic stress responses. For example, PLAT domain-like proteins were linked to tolerance to cold stress in *Allium sativum* [46], and cold and salt stress in *A. thaliana* [47]. Given that in *M. truncatula*, the 14-3-3 protein is distributed across various plant tissues [42], here we initially examined the tissue-specific expression of *MsGRF2* in alfalfa. Our findings revealed the highest *MsGRF2* expression in inflorescences and the lowest in below-ground tissues (Figure 8A), which aligns with expression patterns seen in other crops [42], and suggests that *MsGRF2* may differentially regulate growth and development across tissues [23]. We then investigated the expression patterns of *MsGRF2* under cold, drought, and salt stress conditions to clarify its potential role in abiotic stress responses. The results demonstrated that *MsGRF2* expression significantly decreased under low temperatures. In addition, no significant changes in *MsGRF2* expression were detected under salt stress. These results suggest that *MsGRF2* does not contribute to cold and salt stress tolerance. In contrast, in response to drought, *MsGRF2* expression initially declined but subsequently increased, indicating a drought-induced response similar to patterns observed in other plant species [48,49]. Collectively, these results indicate that *MsGRF2* may play a significant role in alfalfa drought tolerance. To further investigate the role of *MsGRF2* under cold, salt, and drought stress, we introduced this gene into tobacco plants and assessed the stress responses of the resulting transgenic lines. The results revealed significant physiological differences between transgenic plants and WT, especially under drought conditions, indicating that *MsGRF2* enhances drought tolerance. To elucidate the mechanisms underlying *MsGRF2*-mediated stress tolerance, we evaluated several physiological indicators, focusing on the role of *MsGRF2* in modulating oxidative stress and ROS detoxification. Active oxygen compounds are known to be highly toxic to plants [50], thus we measured relevant oxidoreductase activity indicators, including MDA, which reflects membrane lipid peroxidation [51]. The results revealed that MDA levels did not differ significantly between transgenic and WT plants, suggesting that *MsGRF2* may not engage directly in lipid antioxidation pathways [52]. However, proline, a compound associated with stress regulation, was significantly increased under cold and drought stress conditions, implicating *MsGRF2* in proline-related stress responses. SP and SS levels showed variable responses across cold and drought stress, likely reflecting differential 14-3-3 protein expression in response to distinct stressors. Additionally, POD and SOD levels were markedly higher in transgenic plants under drought, indicating potential *MsGRF2* involvement in ROS scavenging. Superoxide anion levels were reduced under normal conditions but remained similar to WT levels under stress, possibly due to the signaling function of superoxide anions during stress responses [53]. Overall, physiological assessments under cold, salt, and drought stress indicated that MsGRF2 primarily enhances drought tolerance.

To investigate the mechanisms by which *MsGRF2* modulates drought stress tolerance, we analyzed the expression of stress-related genes in both WT and transgenic tobacco lines under drought stress treatments. The BRI1 signaling pathway promotes the phosphorylation of plasma membrane (PM) H^+^-ATPases, with 14-3-3 proteins known to enhance the catalytic activity of these H^+^-ATPases [54,55]. Under control conditions, transgenic tobacco plants exhibited elevated *BRI1* expression, suggesting enhanced H^+^-ATPase phosphorylation. However, drought stress reduced *BRI1* expression, diminishing H^+^-ATPase phosphorylation and increasing apoplast pH, which has been shown to improve plant stress resistance [56], suggesting that *MsGRF2* overexpression under drought conditions improves drought tolerance by down-regulating *BRI1*. The results indicate that *Cu*/*Zn-SOD* and *Mn-SOD*, key genes encoding superoxide dismutases, were downregulated in transgenic plants under drought stress, despite their upregulation under non-stress conditions. Under control conditions, transgenic plants exhibited high baseline levels of *Cu*/*Zn-SOD* and *Mn-SOD* expression, suggesting a pre-established, robust antioxidant defense system that minimizes the necessity for further up-regulation under drought conditions. In contrast, WT plants rely on up-regulating these genes in response to drought as a mechanism to alleviate oxidative stress. These results highlight that *MsGRF2* overexpression provided enhanced baseline antioxidant protection against drought stress in transgenic plants. Ethylene plays a regulatory role in the mechanism of 14-3-3 proteins, with *EIL1* and *ERF2* being key genes involved in its biosynthesis [44,57]. Under non-stress conditions, *EIL1* expression was elevated in transgenic plants, likely as a result of MsGRF2 overexpression, which increases 14-3-3 protein levels and subsequently promotes ethylene biosynthesis. However, under drought conditions, excessive ethylene production can impair stress tolerance [58,59]. To mitigate excessive ethylene, *EIL1* expression in transgenic plants was kept at levels comparable to WT through *MsGRF2*-mediated downregulation of *ERF2*, effectively minimizing ethylene’s negative impact on drought stress tolerance.

The GR1 gene, encoding glutathione reductase [60], showed similar expression levels under drought and control conditions, suggesting that *MsGRF2* may not play a role in the glutathione cycle pathway. In contrast, *ERD10B*, which encodes drought-responsive dehydrins, was highly expressed under drought conditions, indicating that *MsGRF2* may enhance drought tolerance by upregulating *ERD10B* [61,62]. *TIP* encoding aquaporins [63] also showed increased expression under stress, leading to higher aquaporin levels that improve water balance and confer drought tolerance; thus, *MsGRF2* likely boosts drought tolerance through *TIP* upregulation. *LTP1*, encoding a lipid transfer protein that stabilizes cell membranes [64,65], showed minimal expression changes under drought, similar to MDA levels, suggesting *MsGRF2* may not enhance drought resistance via membrane structure regulation. *SnRK2*, a key ABA-responsive regulatory factor [66], maintained high expression levels under both drought and control conditions, implying that *MsGRF2* may enhance drought tolerance by activating *SnRK2* and thus facilitating ABA-mediated stress responses [67,68]. Additionally, *KC1*, encoding K^+^ channels involved in pH modulation across membranes, was significantly downregulated in transgenic plants compared to WT, indicating that *MsGRF2* may contribute to drought tolerance by reducing KC1 expression and stabilizing cellular pH under stress [69,70]. A similar mechanism appears to regulate *TPK1*, a two-pore K^+^ (TPK) channel gene, which facilitates cellular homeostasis by modulating K^+^ concentrations [71]. While *TPK1* expression increased under control conditions, it was downregulated under drought, suggesting *MsGRF2* may enhance drought tolerance by modulating K^+^ transport through *TPK1*. Taken together, this study revealed that *MsGRF2* overexpression promoted the expression of stress-related genes and activates multiple pathways—such as ABA signaling, apoplast PH regulation, and ROS detoxification—while concurrently moderating ethylene signaling to enhance drought tolerance. Due to the complexity of plant stress-response regulation, further studies are needed to fully elucidate the specific roles of GRF2 proteins in stress adaptation.

## 4. Materials and Methods

### 4.1. Plant Materials and Treatments

Alfalfa (*Medicago sativa* L. cv. Longmu 801) seeds were disinfected and evenly distributed on moist filter paper in Petri dishes, then incubated in a growth chamber at 25 °C in the dark. After 5 days, seedlings were transplanted into vermiculite-filled plastic pots in a greenhouse and watered every two days with 1/2 strength Hoagland nutrient solution [72], and maintained under a 16/8 h light/dark photoperiod (25/20 °C) with 55–70% relative humidity. Tobacco seeds (K326) were similarly disinfected and uniformly placed on culture dishes containing MS medium (pH 5.8) with 0.8% agar and 15 g/L sucrose, then incubated at 25 °C under a 16 h/8 h light/dark photoperiod.

### 4.2. Identification of GRF Gene Family Members and Analysis of Physical and Chemical Properties

The *Arabidopsis* 14-3-3 protein sequence (n = 13) was retrieved from the TAIR database (https://www.arabidopsis.org/, accessed on 20 June 2024) and subjected to BLAST analysis against the alfalfa Xinjiang Daye macrophyte genome using TBtools. The 14-3-3 specific structural domain 14-3-3 (PF00244) was also retrieved from the Pfam database (http://pfam.xfam.org/, accessed on 22 April 2024) and used in a Hidden Markov Model (HMM) search of the alfalfa genome. The results from both BLAST- and HMM-based searches were analyzed using the TBtools, proteins identified by both methods were considered candidate members of the MsGRF gene family. Physical and chemical properties of MsGRF gene family members were then investigated using the ExPASY platform (https://web.expasy.org/protparam/, accessed on 20 June 2024).

### 4.3. MsGRF Gene Family Member Analyses

The Arabidopsis 14-3-3 gene sequence from the TAIR database was used alongside the *M. truncatula* 14-3-3 protein sequence identified by Qing et al. [42] to construct a phylogenetic tree using neighbor-joining methods in the MEGA7, with 400 bootstrap replicates for robustness. The structural domains within the *MsGRF* gene family were identified via NCBI-CDD platform (https://www.ncbi.nlm.nih.gov/Structure/bwrpsb/bwrpsb.cgi, accessed on 14 June 2024), while conserved motifs were identified using the MEME platform (https://meme-suite.org/meme/tools/meme, accessed on 11 June 2024), and visualized with TBtools.

### 4.4. Gene Sequence Analysis

Total RNA was isolated from untreated alfalfa seedlings using the Ultrapure RNA Kit (CWBIO, Beijing, China), and RNA purity and concentration were assessed with a NanoPhotometer (Implen, Munich, Germany). First-strand complementary DNAs (cDNAs) were synthesized using the HiScript II 1st Strand cDNA Synthesis Kit (+gDNA wiper) (Vazyme, Nanjing, China) following manufacturer’s instructions. cDNAs were subsequently PCR-amplified (A200, LongGene, Hangzhou, China) using primers listed in Figure 4 to obtain amplified *MsGRF2* CDS sequences.

The DNAMAN 6.0.3.99 software was used for amino acid sequences prediction and multiple sequence analysis. The amplified nucleotide sequences and predicted amino acid sequences were compared to available homologs in the NCBI database (https://www.ncbi.nlm.nih.gov/, accessed on 26 March 2021). Phylogenetic analysis of GRF2 protein sequences was conducted using MEGA 7.0 with neighbor-joining methods. Conserved protein domains were identified through the SMART platform (http://smart.embl.de/smart/set_mode.cgi?GENOMIC=1, accessed on 30 March 2021), and Protparam (http://expasy.org/Proteomics, accessed on 30 March 2021) was used to analyze the physical and chemical properties of polygenes. Secondary protein structures were predicted using the SPOMA (http://pbil.ibcp.fr, accessed on 30 March 2021), while three-dimensional structural analysis of the MsGRF2 protein was performed via the SWISS-MODEL platform (https://swissmodel.expasy.org/, accessed on 30 March 2021).

### 4.5. Gene Expression Analysis

Four weeks after transplantation, healthy alfalfa seedlings of similar size were divided into three groups and subjected to cold, drought, and salt stress for 0 (control), 3, 6, 12, 24, and 48 h to evaluate *MsGRF2* expression in response to abiotic stresses. For the cold treatment, alfalfa seedlings were placed in a growth chamber at 4 °C. In the drought- and salt-stress treatments, the alfalfa plants were transferred into nutrient solutions containing 20% polyethylene glycol 6000 (PEG-6000) and 150 mM NaCl, respectively. Roots and leaves from all plants were harvested and immediately frozen in liquid nitrogen, followed by storage at −80 °C. For tissue-specific expression analysis of *MsGRF2*, root, leaf, and inflorescence tissues were collected from six-week-old seedlings. Three biological replicates were used for each treatment, and *MsGRF2* expression across different tissues and stress conditions was quantified using qRT-PCR (q225MX-400, Kubotech, Beijing, China).

### 4.6. Subcellular Localization Analysis of MsGRF2

Five-leaf stage tobacco (*N. benthamiana*) plants were selected for gene transformation experiments, where the vectors 35S::MsGRF2-sGFP and 35S::sGFP were introduced into *Agrobacterium tumefaciens* strain EHA105 to enable transient expression in tobacco leaves. Following transformation, plants were incubated in darkness for 2–3 days to enhance expression stability, after which fluorescence signals were examined using a laser confocal microscope (TCS SP8, Leica, Germany) to confirm successful expression and localization of the GFP-tagged MsGRF2 protein.

### 4.7. Tobacco Transformation and Identification

The pCAMBIA1300 (Preserved in Heilongjiang Provincial Key Laboratory of forage Germplasm Resources and Breeding) vector, containing the 35S promoter was digested using EcoRI and HindIII to construct the pCAMBIA1300-MsGRF2 vector. Vector resistance was conferred by hygromycin (hyg). The resulting construct was transformed into *A. tumefaciens* strain GV3101 (Weidi, Shanghai, China). Transgenic tobacco plants were then generated through the *Agrobacterium*–mediated leaf disc transformation method [73]. Transformants were verified by PCR amplification (A200, LongGene, Hangzhou, China) using specific primer pairs (Table S4).

### 4.8. Transgenic Tobacco Physiological Parameters Measurement

To investigate antioxidant enzyme activity, approximately 0.1 g of fresh leaves from each sample were pulverized in a sodium phosphate buffer containing 1% polyvinyl pyrrolidine. The mixture was then centrifuged for 15 min at 4 °C and 10,000 rpm. The activities of peroxidase (POD) and superoxide dismutase (SOD) were measured at 470 nm and 560 nm, respectively, using a previously described method [74]. Malondialdehyde (MDA) content was measured using a modified thiobarbital acid (TBA) method l [75], while O_2_^−^ concentrations were measured using Elstner’s method [76]. Soluble protein (SP) content was measured using the Bradford assay [77], and reduced glutathione (GSH) concentrations were estimated using a fluorescence-based method [78]. Proline (Pro) content was determined using the ninhydrin method [79] and soluble sugar (SS) concentrations were determined using the Dreywood method [80].

### 4.9. Gene Expression After Stress Exposure

After germination, uniformly developed tobacco seedlings were grown for one month in a greenhouse before exposure to cold, drought, or salt-stress conditions. Leaf samples were collected on the fifth day of each treatment, and immediately frozen at −80 °C for subsequent analysis. For the drought condition treatments, the expression of the stress-related genes *BRI1*, *Cu*, *Zn-SOD*, *Mn-SOD*, *EIL1*, *ERD10B*, *ERF2*, *GR1*, *LTP1*, *KC1*, *SERK3B*, *SnRK2*, *TIP*, and *TPK1* were analyzed with *NtActin* as the internal housekeeping gene.

### 4.10. Statistical Analyses

Each experiment included three independent replicates, from which the mean values and standard deviations were calculated. Statistical differences were evaluated using either one-way analysis of variance (ANOVA) or Student’s *t*-tests in SPSS 19.0, with significance levels designated as follows: *: *p* < 0.05; **: *p* < 0.01. Mapping was performed with Origin 2021.

## 5. Conclusions

In this study, 66 14-3-3 (GRF) family genes encoding 86–308 amino acids were identified in alfalfa, classified into ε- and non-ε groups, and mapped across 19 chromosomes. Promoter regions for these genes were enriched in hormone-responsive and stress-related cis-elements. The *MsGRF2* gene was expressed in all tissues, with particularly high expression in flowers, suggesting tissue-specific roles. Experimental analysis demonstrated that *MsGRF2* contributes to drought resistance by altering the expression of stress-related genes. The results provide a theoretical foundation for evaluating drought resistance mechanisms in alfalfa and breeding of new drought-resistant varieties, which can help improve alfalfa yields under drought stress. Future research should explore the specific molecular interactions between MsGRF2 and its target proteins under drought conditions to better understand the signaling pathways involved in drought tolerance. Additionally, investigating the regulatory roles of other 14-3-3 family members in alfalfa may reveal synergistic effects that further enhance stress tolerance.

## Data Availability

The data presented in this study are openly available in NCBI.

## Supplementary Materials

The following supporting information can be downloaded at: https://www.mdpi.com/article/10.3390/ijms252212304/s1.
