# Supplementary material for: Functional Characterization of the 14-3-3 Gene Family in Alfalfa and the Role of MsGRF2 in Drought Response Mechanisms
Source: Int J Mol Sci. 2024 Nov 16;25(22):12304. doi: 10.3390/ijms252212304 (PMC11595020; doi:10.3390/ijms252212304)
Supplement: Supplementary file 1 [file ijms-25-12304-s001.zip › ijms-3283912-supplementary.pdf]

International Journal of Molecular Sciences

MDPI

Supplementary Materials

Table S1. Physical and chemical property analysis of GRF family in *M. sativa*

Gene name	Protein[a]	MW[b]	pI[c]	II[d]	AI[e]	GRAVY[f]
MsGRF1	111	12853.51	4.72	55.79	80.81	−0.48
MsGRF2	252	28437.04	4.68	44.32	90.6	−0.37
MsGRF3	261	29524.93	4.76	41.5	77.82	−0.643
MsGRF4	263	30122.8	4.76	44.23	80.87	−0.598
MsGRF5	285	32386.11	4.78	45.92	79.47	−0.546
MsGRF6	261	29524.93	4.76	41.5	77.82	−0.643
MsGRF7	263	30122.8	4.76	44.23	80.87	−0.598
MsGRF8	157	17966.54	7.68	30.34	91.78	−0.297
MsGRF9	285	32386.11	4.78	45.92	79.47	−0.546
MsGRF10	261	29524.93	4.76	41.5	77.82	−0.643
MsGRF11	263	30122.8	4.76	44.23	80.87	−0.598
MsGRF12	152	17338.71	5.29	27.86	94.8	−0.294
MsGRF13	258	29148.55	4.74	45.6	81.74	−0.553
MsGRF14	261	29524.93	4.76	41.5	77.82	−0.643
MsGRF15	157	17943.34	5.12	31.44	89.94	−0.332
MsGRF16	285	32386.11	4.78	45.92	79.47	−0.546
MsGRF17	260	30281.39	5.04	46.07	90.04	−0.487
MsGRF18	137	15858.15	5.81	54.73	91.75	−0.451
MsGRF19	260	30164.33	5.25	47.42	91.15	−0.461
MsGRF20	260	30225.35	4.89	51.41	90.77	−0.473
MsGRF21	216	25028.71	5.4	45.45	94.81	−0.315
MsGRF22	260	29209.78	4.7	44.98	83.81	−0.476
MsGRF23	232	26496.88	5.31	41.02	72.67	−0.396
MsGRF24	253	29372.41	5.1	45.74	90.99	−0.439
MsGRF25	260	29339.8	4.71	49.22	83.08	−0.556
MsGRF26	252	28437.04	4.68	44.32	90.6	−0.37
MsGRF27	260	30276.37	5.05	48.39	90.04	−0.509
MsGRF28	232	26755.39	4.89	41.37	89.96	−0.376
MsGRF29	260	30163.45	5.17	43.46	94.54	−0.43
MsGRF30	260	29339.8	4.71	49.22	83.08	−0.556
MsGRF31	252	28437.04	4.68	44.32	90.6	−0.37
MsGRF32	260	30279.42	5.05	45.1	90.42	−0.487
MsGRF33	260	30261.27	4.9	44.36	90	−0.499
MsGRF34	208	23914.29	5.03	43.06	86.35	−0.307
MsGRF35	260	30163.45	5.17	43.46	94.54	−0.43
MsGRF36	86	9839.41	5.17	55.32	89.65	−0.173
MsGRF37	248	27975.08	4.71	42.07	82.26	−0.568
MsGRF38	249	28402.17	4.96	49.22	84.3	−0.471
MsGRF39	224	25355.8	5.07	46.78	89.33	−0.363
MsGRF40	131	15089.25	5.94	41.3	83.36	−0.549
MsGRF41	232	26279.64	4.77	49.83	82.89	−0.475
MsGRF42	135	15591.64	6.15	31.05	83.7	−0.576
MsGRF43	248	27975.08	4.71	42.07	82.26	−0.568
MsGRF44	260	29627.42	4.75	51.13	79.96	−0.52

MsGRF45	135	15503.49	6.15	32.96	82.3	−0.621
MsGRF46	240	25621.58	5.52	42.95	73.62	−0.245
MsGRF47	264	29687.34	4.79	46.58	84.39	−0.465
MsGRF48	308	33345.5	5.97	44.75	96.69	−0.179
MsGRF49	258	29367.08	4.84	53.61	79.11	−0.584
MsGRF50	259	29504.22	4.89	53.15	78.8	−0.595
MsGRF51	260	29193.78	4.7	44.98	84.19	−0.457
MsGRF52	259	29504.22	4.89	53.15	78.8	−0.595
MsGRF53	259	29504.22	4.89	53.15	78.8	−0.595
MsGRF54	260	29209.78	4.7	44.98	83.81	−0.476
MsGRF55	259	29504.22	4.89	53.15	78.8	−0.595
MsGRF56	260	29193.78	4.7	44.98	84.19	−0.457
MsGRF57	260	29193.78	4.7	44.98	84.19	−0.457
MsGRF58	111	12779.43	4.7	59.75	82.61	−0.406
MsGRF59	135	15503.49	6.15	32.96	82.3	−0.621
MsGRF60	130	14770.6	5.75	27.65	85.46	−0.631
MsGRF61	248	27975.08	4.71	42.07	82.26	−0.568
MsGRF62	248	27975.08	4.71	42.07	82.26	−0.568
MsGRF63	252	28437.04	4.68	44.32	90.6	−0.37
MsGRF64	248	27975.08	4.71	42.07	82.26	−0.568
MsGRF65	248	27975.08	4.71	42.07	82.26	−0.568
MsGRF66	260	29193.78	4.7	44.98	84.19	−0.457

[a] Protein (amino acid length, aa); [b]MW (molecular weight, Da); [c]pI (theoretical isoelectric point); [d]II (instability index); [e]AI (aliphatic index); [f]GRAVY (grand average of hydropathicity).

Table S2. Motif composition of 10 *M. sativa* GRF proteins.

	Logo	E-value	Sites	Width
Motif 1	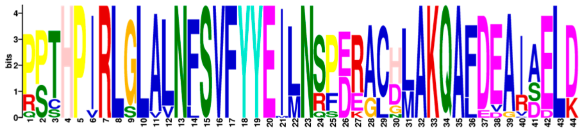	8.9e−1944	50	44
Motif 2	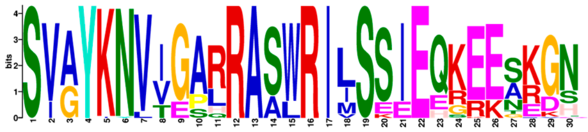	1.4e−1413	62	30
Motif 3	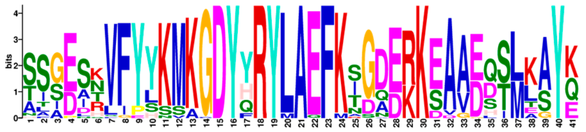	3.3e−1781	61	41
Motif 4	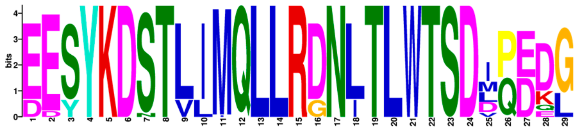	1.2e−1195	48	29
Motif 5	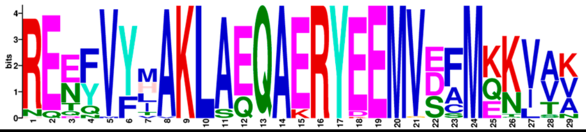	6.5e−1142	54	29

Motif 6	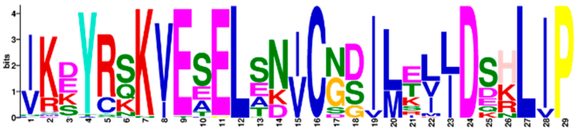	1.6e−1000	52	29
Motif 7	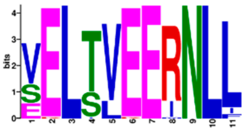	1.3e−416	63	11
Motif 8	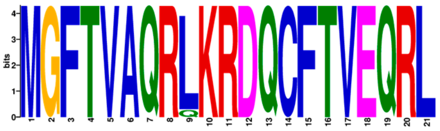	3.3e−224	13	21
Motif 9	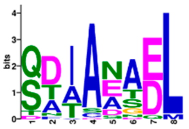	2.1e−113	59	8
Motif 10	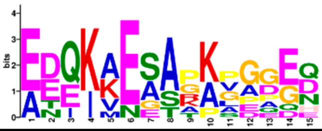	1.6e−093	30	15

Table S3. Ka, Ks analysis of MsGRF genes in *M. sativa*

Duplicate1	Duplicate2	Ka	Ks	Ka/Ks
MsGRF63	MsGRF23	0	0	NaN
MsGRF63	MsGRF26	0	0	NaN
MsGRF63	MsGRF31	0	0	NaN
MsGRF5	MsGRF9	0	0.010292	0
MsGRF3	MsGRF6	0	0.011247	0
MsGRF4	MsGRF7	0	0.034992	0
MsGRF5	MsGRF13	0	0.011289	0
MsGRF3	MsGRF10	0	0.016934	0
MsGRF4	MsGRF11	0	0.017292	0
MsGRF5	MsGRF16	0	0.015491	0
MsGRF3	MsGRF14	0	0.028439	0
MsGRF8	MsGRF12	0.008565	0.009701	0.882938
MsGRF9	MsGRF13	0	0.011289	0
MsGRF6	MsGRF10	0	0.005602	0
MsGRF7	MsGRF11	0	0.017292	0
MsGRF8	MsGRF15	0.022291	0.009427	2.364666
MsGRF9	MsGRF16	0	0.015491	0
MsGRF6	MsGRF14	0	0.016934	0
MsGRF12	MsGRF15	0.020126	0	NaN
MsGRF13	MsGRF16	0	0.016998	0
MsGRF10	MsGRF14	0	0.022665	0
MsGRF22	MsGRF25	0	0.005556	0
MsGRF23	MsGRF26	0	0	NaN
MsGRF17	MsGRF24	0.023318	0.051431	0.453379
MsGRF17	MsGRF27	0.006617	0.005808	1.139235
MsGRF20	MsGRF28	0.015883	0.022903	0.693525
MsGRF21	MsGRF29	0.017239	0.031418	0.548702

MsGRF22	MsGRF30	0	0.011153	0
MsGRF23	MsGRF31	0	0	NaN
MsGRF17	MsGRF32	0.004956	0	NaN
MsGRF18	MsGRF33	0.012679	0	NaN
MsGRF21	MsGRF35	0.017239	0.031418	0.548702
MsGRF25	MsGRF30	0	0.005556	0
MsGRF24	MsGRF27	0.023318	0.057721	0.403978
MsGRF26	MsGRF31	0	0	NaN
MsGRF24	MsGRF32	0.025067	0.051483	0.486898
MsGRF27	MsGRF32	0.008278	0.005814	1.423848
MsGRF28	MsGRF33	0.01778	0.01627	1.092834
MsGRF38	MsGRF39	0.00193	0	NaN
MsGRF38	MsGRF44	0.017926	0.022615	0.792652
MsGRF38	MsGRF53	0.05274	0.643966	0.081898
MsGRF39	MsGRF44	0.021967	0.02503	0.877625
MsGRF44	MsGRF53	0.046294	0.677682	0.068312
MsGRF44	MsGRF55	0.046294	0.649609	0.071264
MsGRF47	MsGRF51	0	0	NaN
MsGRF46	MsGRF48	0.02061	0.00561	3.67375
MsGRF47	MsGRF89	0	0	NaN
MsGRF49	MsGRF86	0	0.005837	0
MsGRF51	MsGRF54	0.001669	0.028305	0.058947
MsGRF49	MsGRF55	0	0.048016	0
MsGRF51	MsGRF56	0	0	NaN
MsGRF53	MsGRF55	0	0.054132	0
MsGRF54	MsGRF56	0.001669	0.028305	0.058947
MsGRF59	MsGRF60	0	0	NaN

Ka = number of SNPs with non-synonymous substitutions / number of non-synonymous substitution sites; Ks = number of SNPs with synonymous substitutions / number of sites with synonymous substitutions

Table S4. The primers used in this study.

Gene	Primers (5'-3')	Notes
Actin-F	TTTGAGACTTTCAATGTGCCCGCC	RT-qPCR
Actin-R	TAGCATGTGGGAGTGCATAACCCT	RT-qPCR
BRI1-F	CTCTGGTAAGTTGCCTGTTGATA	RT-qPCR
BRI1-R	GACTCGGGTAAACCACCAATAA	RT-qPCR
EIL1-F	TCTGAAGAAGGCGTGGAAAG	RT-qPCR
EIL1-R	TTCCTTGGCCGTCATCTTATC	RT-qPCR
ERF2-F	GGCTCCAAGGCTCTATTGAA	RT-qPCR
ERF2-R	GGTTCAGGTGATCGTCTCTTAG	RT-qPCR
Mn-SOD-F	CGGCAATTAGCGGTGACATA	RT-qPCR
Mn-SOD-R	ATGGCGTCATGTAGCTGTTC	RT-qPCR

Cu/Zn-SOD-F	ACTTACACACAGAGCCAGCC	RT-qPCR
Cu/Zn-SOD-R	CGGTGGCAGTACTAGGGATG	RT-qPCR
ERD10B-F	CAAGGCGGAAGAAGGAAGAA	RT-qPCR
ERD10B-R	CGTAGTTGTTGCAGTTGAATGAG	RT-qPCR
GR1-F	CAGAATGACCAGAGTCCTCTAAC	RT-qPCR
GR1-R	CCAGCACTTCATGTTTGTCTTC	RT-qPCR
LTP1-F	CGAATTTGGCACCTTGTCTTG	RT-qPCR
LTP1-R	GCAGAATTCACCAGAGCCTTA	RT-qPCR
SnRK2-F	CTGTAGGGACGCCTGCTTATG	RT-qPCR
SnRK2-R	TGGACTTGTTCGGGGATTGA	RT-qPCR
TPK1-F	GGTCTCTCTCGTGGTGTTAATG	RT-qPCR
TPK1-R	GGTTGTGATGGTAGAACAGACA	RT-qPCR
TIP-F	TCAAGCACCACAGTCATCTC	RT-qPCR
TIP-R	CTGGATCTTTCTTTGCCTTGTG	RT-qPCR
KC1-F	AATGGAGGAGCTAGCAGAAATG	RT-qPCR
KC1-R	TGTGTCCCGGCTTCAATAAC	RT-qPCR
MsGRF2-F	ATGGGTGGTGCGATTCCAG	RT-PCR
MsGRF2-R	TCAAGGCTCATCTAGCTGGTCC	RT-PCR
HYG-F	CGATTCCGGAAGTGCTTGAC	RT-PCR
HYG-R	CGTCTGCTGCTCCATACAAG	RT-PCR
MsGRF2-F(XbaI)	ggtacccggggatcctctagaATGGGTGGTGCGATTCCAG	pCAMBIA1300-sGFP
MsGRF2-R(XbaI)	gctcaccatgtcgactctagaAGGCTCATCTAGCTGGTCCTGG	pCAMBIA1300-sGFP
MsGRF2-F(EcoRI)	tatgaccatgattacgaattcATGGGTGGTGCGATTCCAG	pCAMBIA1300
MsGRF2-R(HindIII)	acgacggccagtgccaagcttTCAAGGCTCATCTAGCTGGTCC	pCAMBIA1300

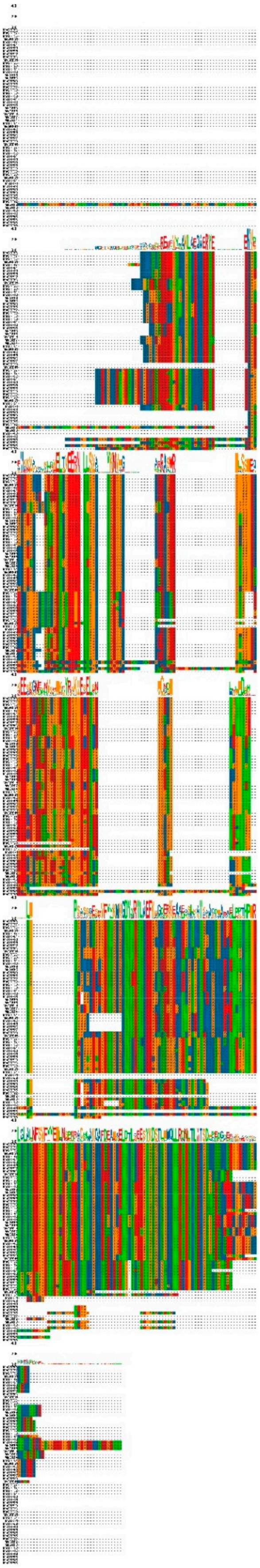

Figure S1. Alignment of MsGRFs protein sequence in *M. sativa*

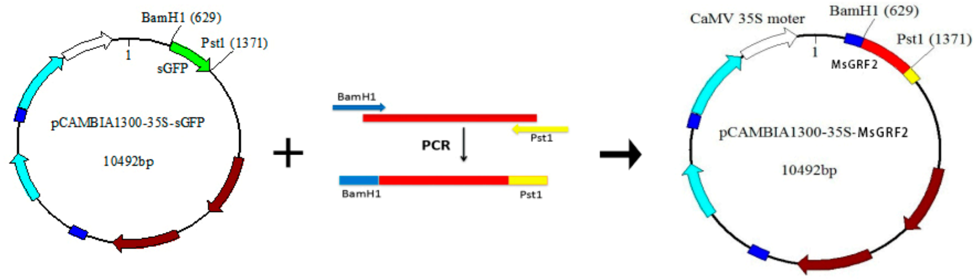

Figure S2. Construction of plant expression vector *35S::MsGRF2-sGFP*

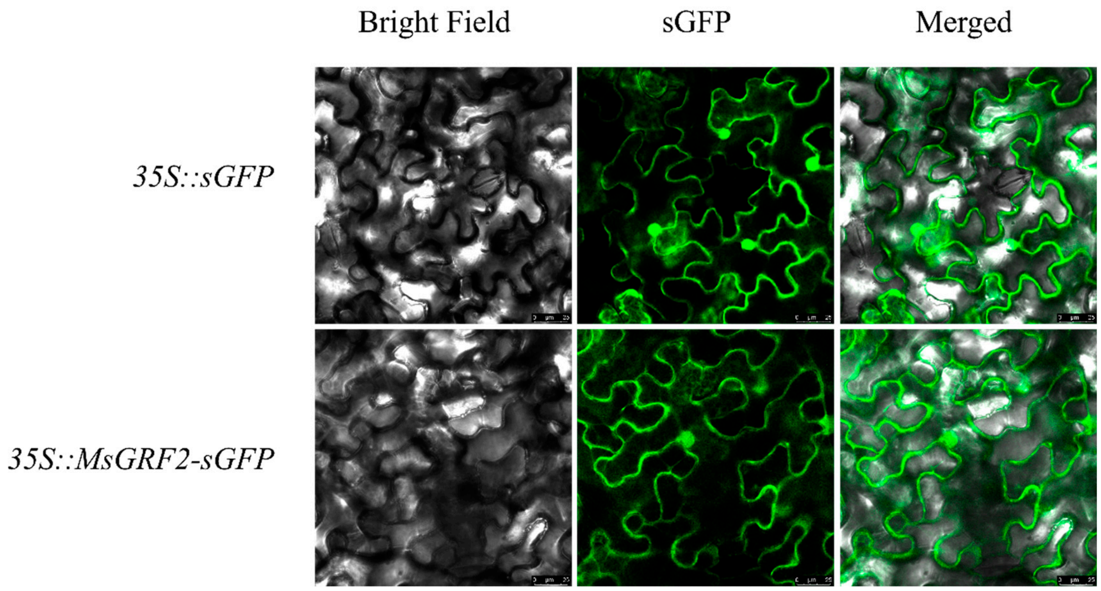

Figure S3. Subcellular localization of MsGRF2 protein. The *35S::MsGRF2-sGFP* or *35S::sGFP* alone fusion constructs were expressed in tobacco epidermal cells and visualized using a confocal fluorescence microscope. Scale bar, 25 μm.
